# Impact of antigen retrieval protocols on the immunohistochemical detection of epigenetic DNA modifications

**DOI:** 10.1007/s00418-023-02187-4

**Published:** 2023-04-03

**Authors:** Jobran M. Moshi, Monique Ummelen, Jos L. V. Broers, Frans C. S. Ramaekers, Anton H. N. Hopman

**Affiliations:** 1 Department of Molecular Cell Biology, GROW-School for Oncology and Reproduction, Maastricht University Medical Center, P.O. Box 616, 6200 MD Maastricht, The Netherlands; 2 Department of Medical Laboratory Technology, Faculty of Applied Medical Sciences, Jazan University, Jazan, Kingdom of Saudi Arabia

**Keywords:** Uterine cervix, Squamous epithelium, Cultured cells, Chromosomes, 5-methylcytosine, 5-hydroxymethylcytosine, Immunohistochemistry, Antigen retrieval

## Abstract

This study compares three different pretreatment protocols for the immunohistochemical detection of 5-methylcytosine (5-mC) and 5-hydroxymethylcytosine (5-hmC) in nuclear DNA. The human biological samples analyzed included formalin-fixed and paraffin-embedded (FFPE) normal squamous epithelium, ethanol-fixed cultured cells, and metaphase chromosomes. The antigen retrieval methods included low pH Citrate and high pH Tris–ethylenediaminetetraacetic acid (EDTA) protocols, as well as a method using Pepsin pretreatment combined with HCl for DNA denaturation. A gradual increase in the detection levels of 5-mC and 5-hmC was observed when going from Citrate via Tris/EDTA to Pepsin/HCl retrieval. While the Citrate retrieval protocol was the least efficient for the detection of 5-mC and 5-hmC, it did preserve nuclear morphology and enabled visualization of differences in intra- and internuclear distribution patterns in tissue and cell culture samples by single- and double-fluorescence detection. Quantification of (hydroxy)methylation levels in FFPE material demonstrated a significant heterogeneity and differences in 5-mC and 5-hmC levels within and between nuclei in the different compartments of normal squamous epithelium. It was concluded that immunohistochemical detection of 5-mC and 5-hmC enables the correlation of these DNA modifications with histomorphological features in heterogeneous tissues, but this is influenced by different pretreatment protocols that must be carefully chosen to allow an appropriate interpretation of these epigenetic switches.

**Supplementary Information:**

The online version contains supplementary material available at 10.1007/s00418-023-02187-4.

## Introduction

The methylation of deoxycytidine moieties is one of the major processes for the epigenetic regulation of DNA activity. It plays a critical role in regulating gene activity during fertilization, early embryogenesis and development, X-chromosome inactivation, genomic imprinting, and chromatin organization. Also, alterations in the methylation status impact the activation or inhibition of oncogenes or tumor-suppressor genes. DNA methylation generally turns off specific gene activity, while demethylation induces reactivation and expression of these genes (Paulsen and Ferguson-Smith 2001; Esteller 2007; Handy et al. 2011; Miller and Grant 2013; Moore et al. 2013). DNA methylation occurs predominantly on CpG dinucleotides, with 60–90% of these dinucleotides being methylated in the mammalian genome. One of the most common epigenetic modifications involves DNA methyltransferase, which is responsible for methylation at the 5′ position of cytosine residues of CpG dinucleotides, creating 5-methylcytosine (5-mC) (see, for example, Lu et al. 2020).

Reactivation of epigenetically silenced genes starts with the hydroxylation of 5-mC, resulting in 5-hydroxymethylcytosine (5-hmC). This modification step is initiated by the ten-eleven translocation (TET) enzyme family, which can convert 5-mC to 5-hmC and other cytosine derivatives and, as such, plays a central role in the DNA demethylation process (Tahiliani et al. 2009; Yang et al. 2013; Pfeifer et al. 2014). Furthermore, He et al. (2021) recently published convincing evidence that 5-hmC is a fundamental regulatory element affecting tissue-specific gene expression programs and functions.

Detection methods for gene-specific 5-mC and 5-hmC DNA modifications are mostly based on methylation-specific polymerase chain reaction (PCR) assays or sequencing analyses of isolated DNA (Rein et al. 1998; Storebjerg et al. 2018). The overall hyper- and hypomethylation status of an individual cell can be determined by using antibodies directed against 5-mC and 5-hmC in immunohisto(cyto)chemical procedures applying fluorescence or bright-field microscopy (Singh et al. 2018; Kato et al. 2020). Immunohistochemical studies targeting 5-mC and 5-hmC have been performed on different types of biological samples, including tumor tissues (Szulwach et al. 2011; Singh et al. 2018; Kato et al. 2020). Discrepancies in staining patterns, the presence of positive nuclei adjacent to negative ones, and the impact of immunocytochemical pretreatment protocols on the detection sensitivity of 5-mC and 5-hmC have been reported (Pendina et al. 2017; Wang et al. 2019; Estévez-Torres and Baigl 2011). Furthermore, the detectability of these modified nucleotides in tissues and cells may differ significantly with different DNA and protein compactness in different areas of the nucleus or tissue sample (Estévez-Torres and Baigl 2011), which may result in artificial negative results. In this respect, it is crucial to apply appropriate retrieval methods for optimal availability of the antigenic sites for antibody binding.

The aim of our study was to compare and revisit three pretreatment protocols to (simultaneously) detect 5-mC and 5-hmC. These retrieval protocols are based on widely applied protein retrieval methods such as microwave treatment in a low pH Citrate buffer or a high pH Tris–ethylenediaminetetraacetic acid (EDTA) buffer solution. A third retrieval protocol applied in this study, the Pepsin/HCl pretreatment procedure, was initially developed to detect DNA-incorporated BrdU in formalin-fixed and paraffin-embedded (FFPE) tissues. These pretreatment methods exhibit significant differences in their efficacy of protein removal and exposing DNA, which is needed to enable antibody penetration and binding to the modified nucleotides. Several types of biological materials were analyzed, including FFPE normal squamous epithelium tissue from the uterine cervix, ethanol-fixed cell lines established from cervical cancers (HeLa, CaSki, SiHa), and human metaphase chromosomes from normal lymphocytes and the CaSki cancer cell culture. Special attention was paid to the levels of 5-mC and 5-hmC in the different cellular compartments of the squamous epithelium, including the basal and parabasal (progenitor) cells and the differentiating epithelium. Detection of 5-mC and 5-hmC was performed using single-color bright-field microscopy, while fluorescence detection enabled the simultaneous visualization and quantification of 5-mC and 5-hmC levels.

## Materials and methods

### Tissues and cells

Formalin-fixed and paraffin-embedded (FFPE) tissue samples of normal squamous epithelium of the uterine cervix (*n* = 5) were obtained after colposcopy. Samples were selected from the archives of the Pathology Department of the Maastricht University Medical Center, Maastricht, the Netherlands. Samples were formalin-fixed and paraffin-embedded following a protocol for routine histopathological diagnosis and immunohistochemistry. Research on these tissue samples has been performed in accordance with the Code for Proper Secondary Use of Human Tissue in the Netherlands (http://www.federa.org/, update 2011: Human Tissue and Medical Research: Code of conduct for responsible use) and has been approved by the board of the Maastricht Pathology Tissue Collection at the Maastricht University Medical Centre (Registration Number MPTC 2011-05).

Paraffin sections of 4 µm were collected on Superfrost Plus microscope slides (Thermo Fisher Scientific, Waltham, Massachusetts, USA). They were dewaxed in xylol, rehydrated in a descending alcohol series, and processed further using three different antigen retrieval pretreatment protocols (see below).

Three human papillomavirus (HPV)-infected human epidermoid cervical cancer cell lines (HPV 16-positive CaSki and SiHa cells; HPV18-positive HeLa cells) were purchased from the American Type Culture Collection (ATCC) (Manassas, VA, USA). The cells were maintained in Dulbecco’s modified minimal essential medium (DMEM; Life Technologies/GIBCO BRL, Paisley, Scotland) supplemented with 10% fetal bovine serum (HyClone, ThermoFisher Scientific, Landsmeer, the Netherlands) and gentamycin (Eurovet Animal Health BV, Bladel, the Netherlands). After culturing, the adherent cells were dissociated with a Trypsin/EDTA solution (Merck, T40049, Rayway, NJ, USA), washed twice with phosphate buffered saline (PBS; Janssen Chimica, Beerse, Belgium), centrifuged at 350*g* for 5 min, and the cell pellet was resuspended in 70% ethanol (10^6^ cells/ml) and stored at −20 °C. Cell suspensions (10 µl) were spotted on Superfrost Plus microscope slides and heated at 80 °C for 15 min. After heating, the slides were processed using three antigen retrieval pretreatment protocols (see below).

Metaphase preparations were prepared from the CaSki cell line and from peripheral blood lymphocyte samples. Cell suspensions (10 ml) with 10^6^ cells/ml were added to 100 ml Roswell Park Memorial Institute (RPMI) 1640 Medium supplemented with 10% fetal calf serum, antibiotics and phytohemagglutinin M (Thermo-Fisher, Cat no 10576015). The cells were cultured for 72 h, after which 80 µl of KaryoMax Colcemid Solution (GIBCO 15210-040) was added, incubated for 2 h at 37 °C, and centrifuged for 5 min at 200 relative centrifugal force (rcf). Lymphocytes were treated with a hypotonic solution of 0.075 M KCl for 20–30 min at 37 °C and then prefixed with 3:1 methanol:acetic acid for 5 min. Cells were collected by centrifugation and washed four times with 3:1 methanol:acetic acid. After the final centrifugation step, the supernatant was removed completely and the cell pellet was resuspended in 10 ml of final fixative (3:1 methanol:acetic acid). The cell suspension was stored at −20 °C. Next, 10 µl of the cell suspension was spotted onto Superfrost Plus microscopy slides to obtain the metaphase spreads and heated for 15 min at 80 °C on a plate heater. Thereafter, the slides were processed using three different antigen retrieval protocols (see below).

### Antigen retrieval protocols

We used three different pretreatment steps to visualize 5-methylcytosine-modified and 5-hydroxymethylcytosine-modified nucleotides.

A) For the Citrate protocol, the specimens were boiled in a microwave oven for 10 min in 10 mM Na-Citrate buffer (pH 6.0) and then incubated for 20–30 min at room temperature to cool down.
B) For the Tris/EDTA (TE) protocol, the specimens were boiled for 10 min in 10 mM Tris/HCl, 1 mM ethylenediaminetetraacetic (EDTA) buffer (pH 9.0) in a microwave oven and then incubated for 20–30 min at room temperature to cool down.
C) For the Pepsin/HCl protocol, the specimens were incubated with different Pepsin and HCl concentrations. Cell lines and metaphase preparations were incubated for 15 min in 100 µg Pepsin/ml (Sigma P7000), 0.01 M HCl at 37 °C; FFPE tissue sections were incubated in 2 mg Pepsin/ml 0.1 M HCl). After Pepsin treatment, the specimens were incubated in 2 M HCl at 37 °C for 30 min. Subsequently, slides were dip-washed three times with PBS and with PBS containing 0.05% Tween-20 (PBT) for 5 min each.

### Primary anti 5-mC and 5-hmC antibodies

The following primary antibodies were applied in this study:

A) The mouse monoclonal OptimAb anti 5-methylcytosine (anti 5-mC) (BI-MECY-0100, clone 33D3; Eurogentec, Seraing, Belgium). Clone 33D3 recognizes the modified base 5-methylcytidine in DNA and specifically distinguishes it from its normal DNA base counterpart.
B) The recombinant rabbit monoclonal anti-5-hydroxymethylcytosine antibody (anti 5-hmC) (clone RM236, ab214728; Abcam, Cambridge, UK). The antibody ab214728 reacts to 5-hydroxymethylcytosine in both single-stranded and double-stranded DNA. No cross-reactivity with nonmethylated cytosine and methylcytosine in DNA is reported.

The optimal detection levels of 5-mC and 5-hmC were determined by titration to reach an optimized antibody concentration for the different sample types. These dilutions were in the range recommended by the suppliers and as published in previous reports (Hewitt et al. 2014). As negative controls, the primary antibodies were replaced by PBS and incubation was performed with only the secondary antibody.

### Bright-field microscopy

The FFPE tissue sections, pretreated as described above, were incubated with the primary antibodies mouse monoclonal anti 5-mC diluted 1:500 [OptimAnti-5-Methylcytosine (33D3) Eurogentec BI-MECY-0100)] or rabbit monoclonal anti 5-hmC diluted 1:500 [Recombinant Anti-5-hydroxymethylcytosine antibody (RM236) (ab214728)] at 37 °C for 30 min, washed three times for 5 min with PBT and incubated at 37 °C for 30 min with Biotinylated Horse anti-Mouse immunoglobulin (IgG) (Vector Laboratories, BA-2001 Burlington, Canada) or Biotinylated Goat anti-Rabbit IgG, (Vector Laboratories, BA-1000 Burlington, Canada) applied in a 1:200 dilution in PBT with 5% normal goat serum. After washing twice for 5 min with PBT and once for 5 min with PBS, samples were incubated at 37 °C for 30 min with ABC complex, diluted 50× in PBS (Vector Laboratories, PK-6010 Burlingame, CA, USA). Sections were washed and incubated with freshly prepared diaminobenzidine (DAB) (liquid DAB + substrate chromogen system; DAKO, K3467) for 7 min at room temperature and subsequently washed with MilliQ water. The sections were counterstained with hematoxylin (hematoxylin solution modified according to Gill II, Merck, 1.05175.0500), dehydrated in an ascending ethanol series, and mounted with Entallan (Merck 1.07961.0100).

### Fluorescence microscopy

All three sample types were incubated with the primary antibodies, either separately or simultaneously, at 37 °C for 30 min. The dilutions used for the anti 5-mC were as follows: FFPE tissue sections 1:100, cell lines 1:500, metaphases 1:100. The dilutions used for the anti 5-hmC were 1:100 for all three sample types. The samples were washed three times for 5 min in PBT and incubated at 37 °C for 30 min with the secondary antibodies. The following secondary antibodies were applied:

A) Polyclonal Goat anti-Mouse Ig conjugated with fluorescein isothiocyanate (FITC), 1:100 dilution (Southern Biotech; Goat F(ab')2 Anti-Mouse Ig, Human ads-FITC; cat. no. 1012-02).
B) Polyclonal Goat anti-Rabbit Ig conjugated with FITC, 1:100 dilution (Southern Biotech; Goat Anti-Rabbit Ig, Human ads-FITC; cat. no. 4010-02).
C) Polyclonal Goat anti-Mouse IgG conjugated with Texas Red, 1:100 dilution (Southern Biotech; Goat anti-Mouse IgG, Human ads-TXRD; cat. no.1030-07).
D) Polyclonal Goat anti-Rabbit Ig conjugated with Texas Red, 1:100 dilution (Southern Biotech; Goat anti-Rabbit Ig, Human ads-TXRD; cat. no. 4010–07).

Combinations of Goat anti-Mouse IgG FITC/Goat anti-Rabbit IgG Texas Red and Goat anti-Mouse Texas Red/Goat anti-Rabbit Ig FITC were applied in the double label immunofluorescence studies, and were shown to provide similar distribution patterns in the simultaneous detection of 5-mC and 5-hmC.

All the secondary antibodies were diluted in PBT containing 5% normal goat serum. After washing two times for 5 min with PBT and once for 5 min with PBS, the tissue sections and cell samples were dehydrated and mounted with 90% glycerol in 0.02 M Tris/HCl (pH 8.0), containing 0.02% NaN_3_, 2% 1,4-diazabicyclo[2.2.2]octane (DABCO; Merck), and 0.5 µg/ml 4′, 6-diamidino-2-phenylindole (DAPI; Merck)

### Imaging of bright field and fluorescence immunostaining

The bright-field sections were scanned with a Ventana iScan HT slide scanner (Ventana Medical Systems, Inc. Tucson, Arizona, USA) and semi-quantitatively scored for staining levels of 5-mC and 5-hmC. Images were viewed and selected using Image Viewer Software (Ventana MS).

For non-confocal imaging, the fluorescent signals were recorded with the Metasystems Image Pro System (black and white CCD camera; Sandhausen, Germany) mounted on top of a Leica DM-RE fluorescence microscope, equipped with Texas Red, FITC, and DAPI single bandpass filters for single-color analysis. Images were obtained using an automatic integration time, allowing semi-quantitative evaluation and using the full dynamic range of the camera without signal intensity saturation. For the total fluorescence intensity of 5-mC and 5-hmC, as well as the ratio of fluorescence intensity per nucleus, the blue DNA staining (DAPI image) was used to set the nuclear contour for the measurement. The background was subtracted from the total fluorescence intensity measured. For comparison of the fluorescent intensity between the different specimens, a fixed integration time was applied, set at the automated integration time for the specimen with the highest intensity.

For confocal imaging, the sections were imaged with a laser scanning confocal microscope (Leica SPE confocal) using LAS AF software (Leica Application Suite Advanced Fluorescence, version 2.7.3.9723). Imaging was done in acquisition mode XYZ, with an ACS APO 63.0 × 1.30 oil immersion lens, a gain of 800–1000 V, offset between −0.5% and 0%, format of 1024 × 1024 pixels, speed of 400 Hz, and frame average of 3. For DAPI excitation, a laser wavelength of 405 nm was used. FITC was excited at 488 nm in acquisition mode XYZ (emission filter 500–594 nm). Texas Red was excited at 532 nm (emission filter 575–737 nm). Image J (Schindelin et al. 2012; open-source platform: https://imagej.nih.gov/ij/docs/menus/analyze.html; NIH, Bethesda, Maryland, USA) was used for further image analysis, processing, and merging of the fluorescent images for the reconstruction of the sections. An intensity curve was used to set the background correction. The background was subtracted from the total fluorescence intensity measured. For the integrated fluorescence intensity of 5-mC and 5-hmC, as well as the ratio of fluorescence intensity per nucleus, the blue DNA staining (DAPI image) was used to set the nuclear contour for the measurement (Schindelin et al. 2012).

## Results

### Effect of the pretreatment protocols on the detection of 5-mC and 5-hmC in FFPE tissue sections of normal cervical squamous epithelium.

**Fig. 1:**
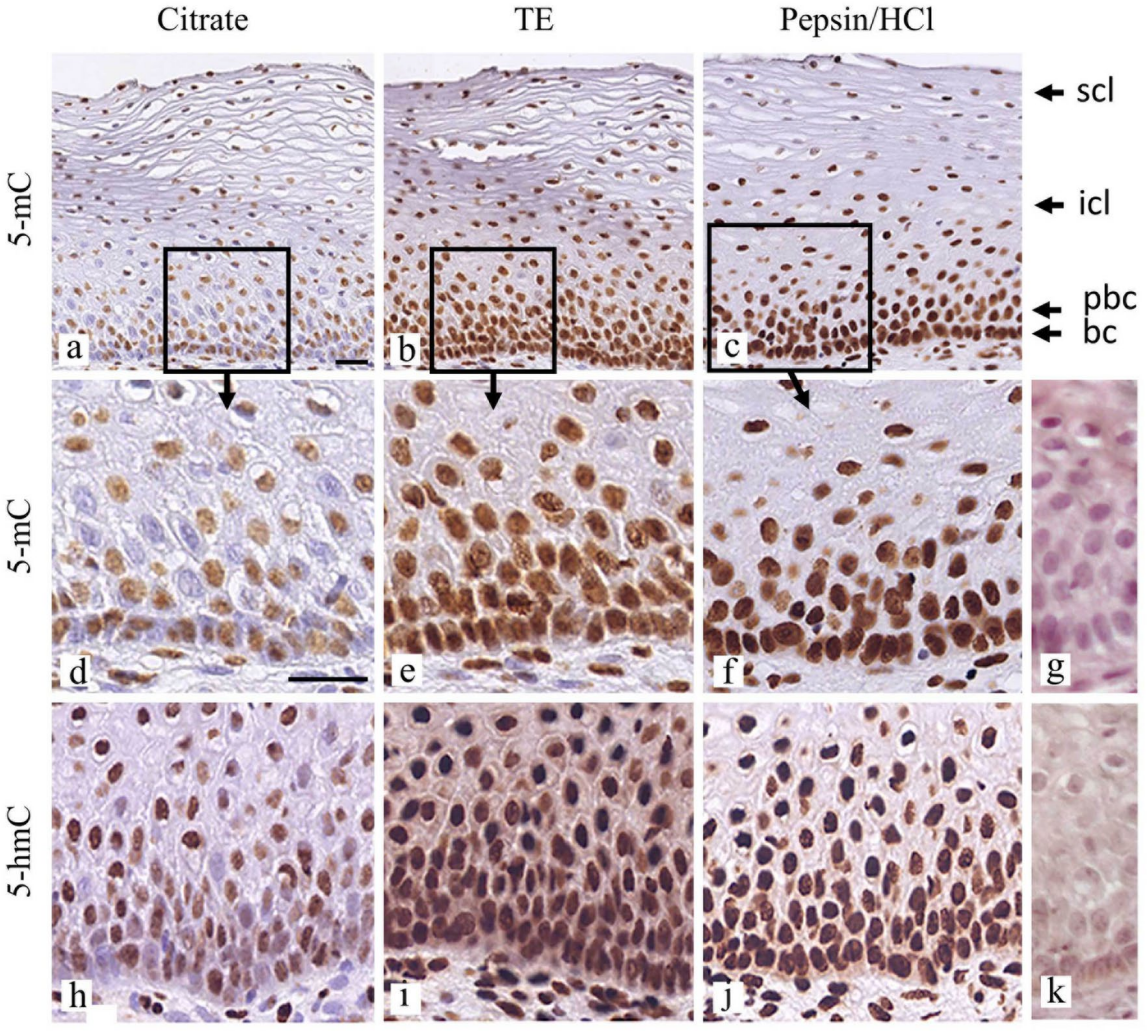
Comparison of retrieval methods to detect 5-mC and 5-hmC in FFPE tissue sections of normal cervical squamous epithelium using bright-field microscopy. **a**–**c** Detection of 5-mC using the Citrate pH 6.0, the Tris/EDTA pH 9.0 (TE), and Pepsin/HCl retrieval methods and a primary antibody dilution of 1:500. **d**–**f** Higher magnifications of the (para)basal compartment as shown in (**a**–**c**). **g** Negative control, primary antibody replaced by PBS, incubation with only the secondary antibody (Pepsin/HCl retrieval). **h**–**j** Detection of 5-hmC using the Citrate pH 6.0, the Tris/EDTA pH 9.0 (TE), and Pepsin/HCl retrieval methods and a primary antibody dilution of 1:500. Same magnifications as in (**d**–**f**). **k** Negative control, primary antibody replaced by PBS, incubation with only the secondary antibody (Pepsin/HCl retrieval). In **a**–**g** and **h**–**k** secondary antibody complexes and dilutions were identical. In **a**, **d**, and **h**, nuclei with a strong staining are seen alternating with nuclei exhibiting a weak or very weak/no staining. In **b**, **c**, **e**, **f**, **i**, and **j**, strongly stained nuclei with slight differences in staining intensity can be recognized. Incidentally, nuclei with weak or no staining can be found. The basal and parabasal cell layers are indicated as bc and pbc, respectively and the intermediate and superficial cell layers by icl and scl, respectively. Scale bars indicate 100 µm in **a** (identical for panels **a**–**c**) and in **d** (identical for panels **d**–**k**)

The effect of the three pretreatment methods on the staining intensities for 5-mC and 5-hmC in FFPE tissue sections of normal squamous cervical epithelium, using bright-field detection and the same primary antibody dilution (1:500) to enable a comparison between the protocols, is illustrated in Fig. 1.

Immunostaining without pretreatment or omission of both primary antibodies did not result in a positive signal (Fig. 1g, k).

A heterogeneous pattern of stained nuclei was recognized when applying the Citrate protocol, with concomitant strongly and weakly immunostained nuclei (Fig. 1a, d, and h). A stronger immunostaining reactivity was observed with the TE and Pepsin/HCl protocol. However, the internuclear staining differences were less obvious and the staining intensities appeared to reach a maximum under the conditions applied (see Fig. 1b, e, and i and Fig. 1c, f, and j for TE and Pepsin/HCl retrieval, respectively). Overall, the normal histological areas in the series of five tissue biopsies studied with the three pretreatment protocols, revealed areas that exhibited this internuclear staining difference, with the most homogeneous and strongest staining intensity being observed with the Pepsin/HCl pretreatment protocol. In addition, with the TE and Pepsin/HCl protocol an increased aspecific background staining was observed when using the 1:500 antibody dilutions. In Fig. 1 the four typical layers that can be recognized in the epithelium are indicated. The basal and parabasal compartment responsible for the renewal of the epithelium, the intermediate layer where the epithelium differentiates, and the terminally differentiating superficial layers that are desquamated into the lumen.

The observation that in these single-label bright-field images a heterogeneous staining pattern was recognized for both 5-mC and 5-hmC in the normal squamous epithelium and stretches of alternating strongly and weakly stained nuclei were recognized in the basal cell compartment of the epithelium, made us wonder whether individual nuclei could exhibit a mutually exclusive staining pattern for 5-mC and 5-hmC, or an overlapping staining reaction for both modifications, or even no staining at all for the two. To study these internuclear differences, we performed both single-label and double-label immunofluorescence microscopy for 5-mC and 5-hmC, and again used the three different retrieval protocols.

**Fig. 2:**
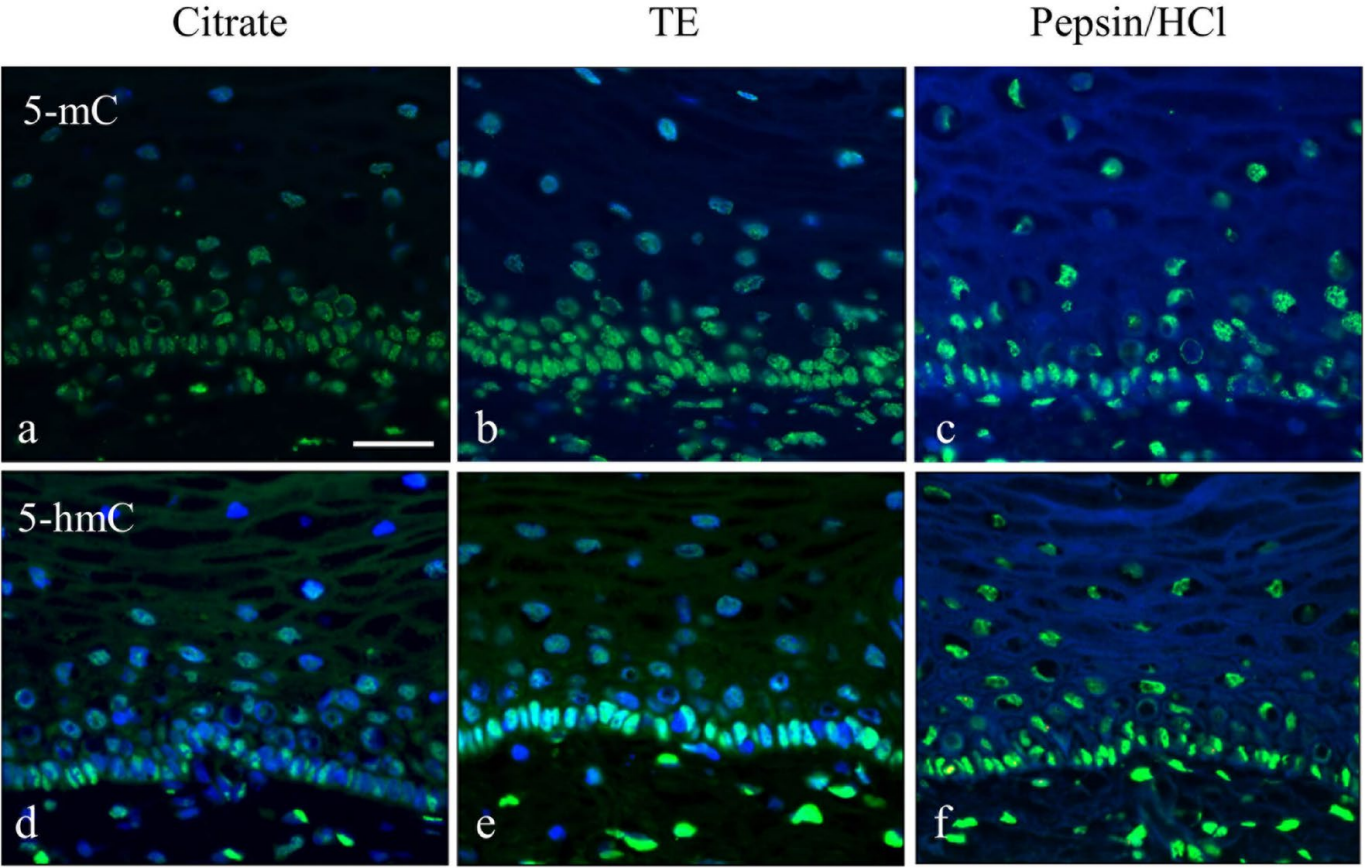
Comparison of three retrieval methods to detect 5-mC and 5-hmC in normal squamous epithelium by single-label immunofluorescence microscopy. **a**–**c** Detection of 5-mC and **d–f** 5-hmC using Citrate pH 6.0, the Tris/EDTA pH 9.0 (TE) and Pepsin/HCl as retrieval method. Primary and secondary antibody dilutions were identical for the detection of 5-mC and 5-hmC and the integration time for collecting images was identical. Both 5-mC and 5-hmC were detected with a FITC (green) labeled secondary antibody and DNA was stained with DAPI (blue). In all images, nuclei with a strong staining alternating with nuclei exhibiting a weak or very weak/no staining can be recognized. Scale bar in **a** indicates 100 µm (identical for panels **a**–**f**)

Using a single-label immunofluorescence approach for 5-mC (Fig. 2a–c) and 5-hmC (Fig. 2d–f) with the three different retrieval methods revealed differences in staining intensities between individual nuclei that ran parallel with the bright-field staining patterns and heterogeneous patterns could again be recognized. In all images, nuclei with a strong staining alternating with nuclei exhibiting a weak or very weak/no staining can be recognized. The Citrate pretreatment resulted in the weakest signal, while the intensity was higher after TE and Pepsin/HCl treatment, as concluded from visual inspection of non-confocal images. The Pepsin/HCl method disabled a proper DAPI intercalation with the DNA and as a result, a poor nuclear staining was obtained, but rather a homogeneous blue background was seen in the slides. Similar results were obtained when using double-label immunofluorescence imaging for the simultaneous detection of 5-mC and 5-hmC in the tissue sections (data for the different retrieval protocols are not shown).

**Fig. 3:**
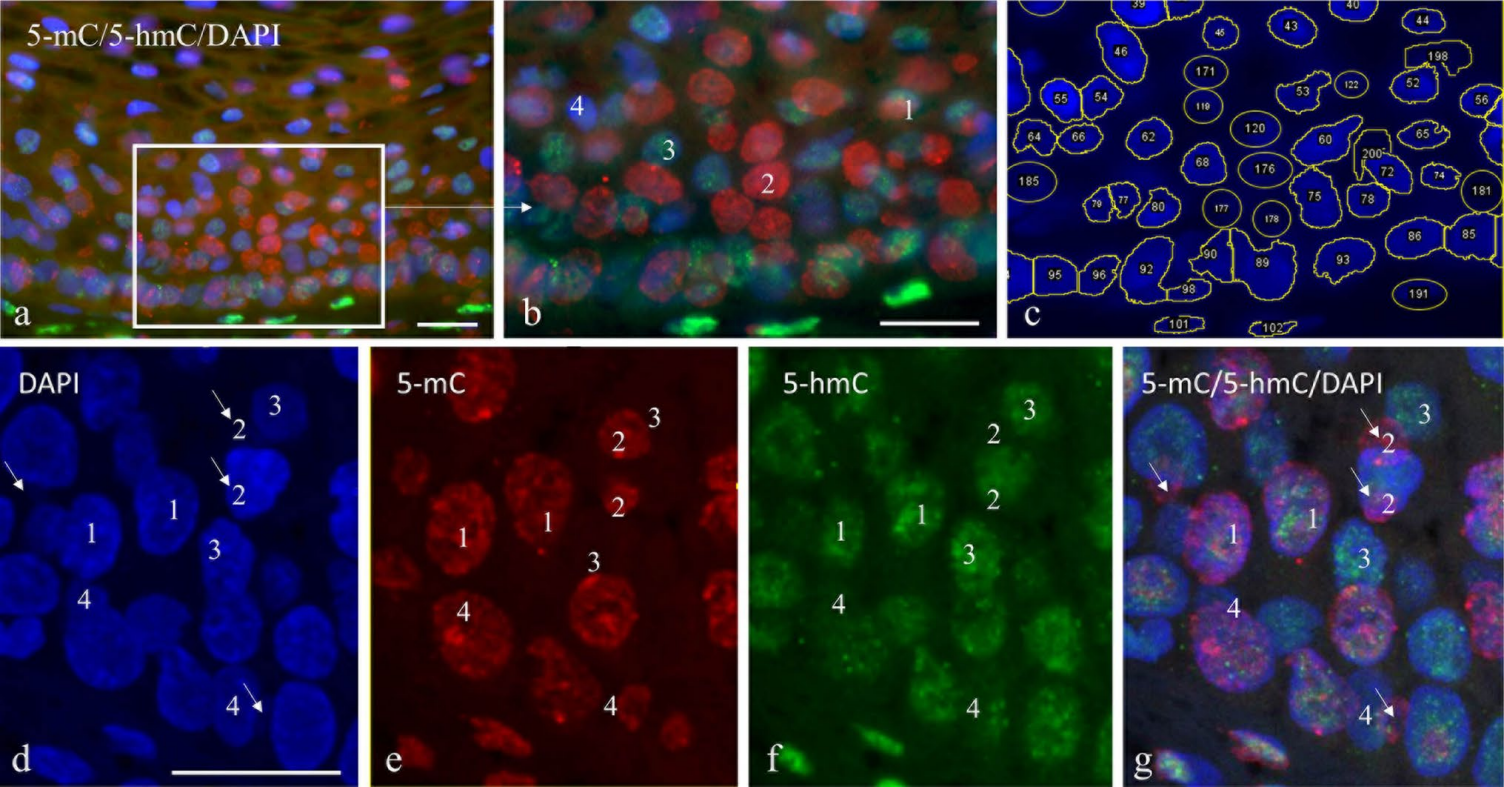
Simultaneous immunofluorescent detection of 5-mC and 5-hmC in normal squamous epithelium, analyzed by non-confocal (**a**–**c**) and confocal microscopy (**d**–**g**) using the Citrate retrieval protocol. **a** 5-mC in red (Texas Red) and 5-hmC in green (FITC) imaged by non-confocal microscopy. **b** Higher magnification of the basal cell compartment as shown in (**a**). **c** Nuclear contours for quantification of immunofluorescence in nuclei as detected by DAPI (blue) staining and determined using ImageJ/Fiji software or manual drawing (see Materials and Methods, Table 1, and Fig. 4). **d**–**g** Imaging by confocal microscopy, showing individual colors for DAPI (**d**), Texas Red (**e**), FITC (**f**), and merged images (**g**). Arrows point to fragments of overlapping nuclei that are out of focus. Numbers in **b** and **d**–**g** refer to the nuclei (or fragments of nuclei) with a particular combination of fluorescence patterns for 5-mC and 5-hmC. (1): strong positivity for both 5-mC and 5-hmC (red and green positive), (2): 5-mC and 5-hmC (red and green), (3): 5-hmC and 5-mC (green and red), and (4): both weak/no staining, only DAPI positive. Scale bars in **a** and **b** indicate 25 µm (identical for panels **b** and **c**) and in **d** (identical for panels **d**–**g**)

Figure 3 shows typical examples of the simultaneous immunofluorescent detection of 5-mC and 5-hmC using the Citrate pretreatment protocol. It may be obvious from the non-confocal microscopy images in Fig. 3a and b, as well as from the confocal images in Fig. 3d–g, that individual nuclei exhibit different combinations of 5-mC and 5-hmC immunostaining patterns. The numbers in Fig. 3b and d–g refer to nuclei with variable fluorescence combinations of Texas Red and FITC fluorescence detecting 5-mC and 5-hmC, respectively: (1): strong positivity for both 5-mC and 5-hmC (red and green positive), (2): 5-mC and 5-hmC (red and green), (3): 5-hmC and 5-mC (green and red), and (4): both weak/no staining for 5-mC and 5-hmC (only DAPI positive). These images clearly illustrate the heterogeneity for the 5-mC and 5-hmC modifications in the different cellular compartments of a heterogeneous tissue.

**Table 1:** Quantification of immunofluorescence intensities (arbitrary units) for 5-mC and 5-hmC in FFPE squamous epithelium samples, pretreated with the three different retrieval protocols and imaged by non-confocal microscopy

Pretreatment	Nr nuclei	5-mC (TxRd)		5-hmC (FITC)		Nr 5-mC/5-hmC negative nuclei
		Nr negative nuclei	Average fluorescence intensity	Nr negative nuclei	Average fluorescence intensity	
Citrate	201	40	14,462	13	17,592	26
	184	17	17,814	2	15,283	4
	123	7	17,742	9	10,902	9
	178	23	20,456	17	25,014	31
	686*	87*	17,504 +	41*	17,699 +	70*
TE	114	4	23,320	2	38,648	3
	157	13	31,107	5	43,131	8
	182	8	51,042	5	60,578	9
	157	12	41,233	2	47,519	3
	610*	37*	38,205 +	14*	48,628 +	23*
Pepsin/HCl	75	nd	81,343	nd	31,911	nd
	107	nd	112,853	nd	67,408	nd
	208	nd	62,859	nd	43,138	nd
	166	nd	62,164	md	52,285	nd
	556*		74,765 +		49,025 +	

For the comparison, identical immunohistochemical detection procedures were followed (including antibody dilutions for primary and secondary antibodies). Images were collected using non-confocal microscopy and a fixed integration time to capture the image was applied (see Materials and Methods). The nuclear contours were determined based on DAPI staining. For the Pepsin/HCl pretreatment, the contour plotting for immunofluorescence measurement was hampered by the weak DAPI staining of the nuclei, so the number of negative nuclei can not be properly assessed. Nuclei with a fluorescence intensity < 5% of the average intensity were not included in the calculation of the average fluorescence intensity per nucleus

*Total number of nuclei; +  average fluorescence intensity per nucleus in the four tissue samples analyzed; *nd* not determined

### Quantification of 5-mC and 5-hmC immunofluorescence in normal squamous epithelium

For the quantitative comparison of the immunostaining levels obtained with the different retrieval protocols, the immunofluorescence intensities for 5-mC and 5-hmC were measured within the DAPI contours as illustrated in Fig. 3c. Table 1 summarizes the average fluorescence intensities per nucleus for 5-mC and 5-hmC, measured using non-confocal imaging in four tissue areas for each pretreatment protocol. The average fluorescence intensity for 5-mC increased gradually from Citrate via TE to Pepsin/HCl retrieval. With Pepsin/HCl, the fluorescence intensity is a factor of five times higher as compared with Citrate pretreatment. For 5-hmC, the difference in average intensity between Citrate and TE/Pepsin/HCl is a factor of about 3.5.

**Fig. 4:**
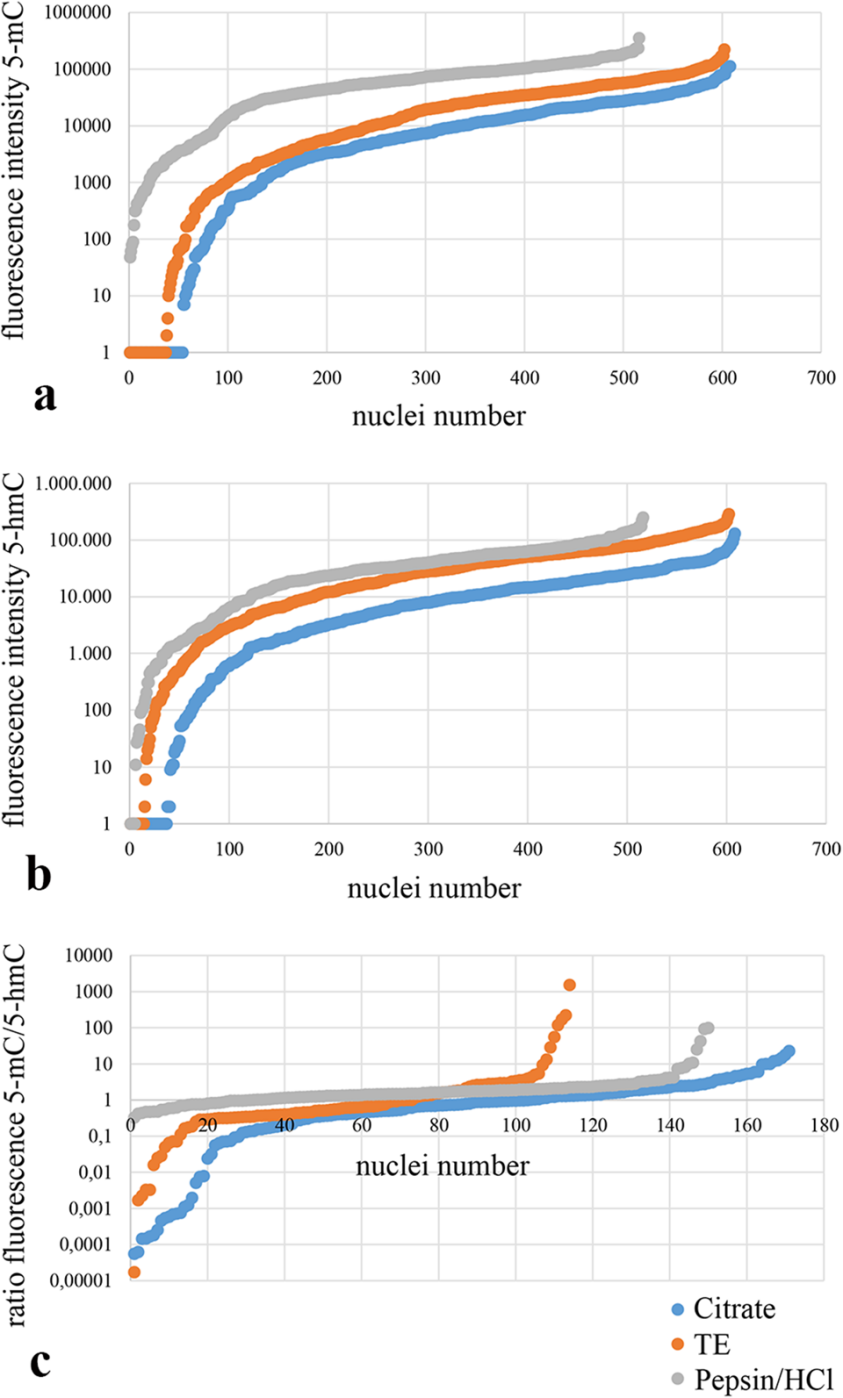
Quantitative immunofluorescence analyses of 5-mC and 5-hmC in nuclei of squamous epithelium tissue sections, using the three different retrieval methods. **a**, **b** Fluorescence intensity measurements of 5-mC (**a**) and 5-hmC (**b**) in a total of 500–600 nuclei (four section areas) on the basis of contour bordering using DAPI (see Fig. 3c). Intensities are displayed on a log scale to enable coverage of a broad range of intensities. In charts **a** and **b** the cell nuclei are lined up from left to right based on increasing fluorescence intensity and the corresponding intensity values are plotted on the *y* axis. **c** Ratio profile of measured intensities for 5-mC and 5-hmC. The ratio of fluorescence per nucleus is plotted on the *y* axis on a log scale to enable comparison of values > 1 and values in the interval between 1 and 0. Nuclei numbers on the *x* axis are lined up from left to right based on the increasing ratio of the 5-mC/5-hmC fluorescence intensity. Blue curve: Citrate protocol; orange curve: TE protocol; gray curve: Pepsin/HCl protocol

The distributions of the fluorescence signal intensities measured in the individual nuclei for 5-mC and 5-hmC are plotted in Fig. 4a and b. These curves illustrate the distribution of nuclear intensities for the three pretreatment protocols. For all protocols, a wide range of intensities was observed, the widest distribution being obtained with Citrate and TE retrieval and the narrowest with Pepsin/HCl. When measured by confocal imaging, similar distribution curves were obtained (data not shown).

To substantiate the internuclear differences as shown in Fig. 3, the ratios between the intensities for 5-mC and 5-hmC were measured per individual nucleus. In Fig. 4c, the ratio distribution measured in individual nuclei is plotted, which clearly illustrates that within the squamous epithelium 5-mC and 5-hmC levels are heterogeneously distributed. These curves show the nuclei with a dominant 5-hmC (5-hmC > 5-mC) staining in the left part of the distribution curve (compare Fig. 3, nuclei indicated with number 3) and nuclei with a dominant 5-mC (5-mC > 5-hmC) staining in the right part of the curve (compare Fig. 3, nuclei marked with number 2). In the middle part of the curve, the intensities are more balanced (compare Fig. 3, nuclei marked with number 1).

### Effect of the pretreatment protocols on 5-mC and 5-hmC detection in cervical cancer cell lines

**Fig. 5:**
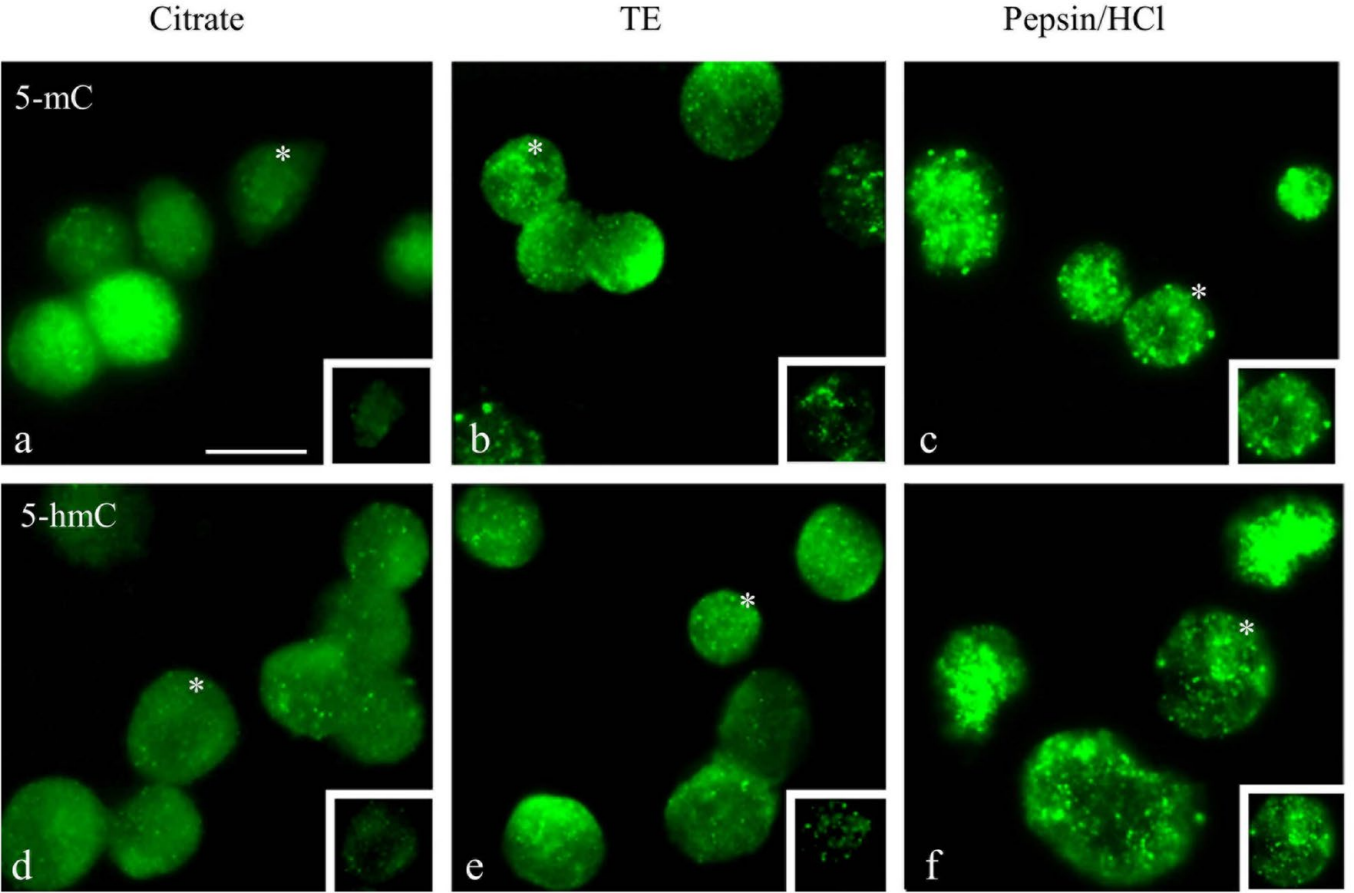
Comparison of fluorescent staining patterns of 5-mC and 5-hmC in CaSki cells using the Citrate, TE, and Pepsin/HCl retrieval methods. **a**–**f** Visualization of 5-mC and 5-hmC (both in green) by non-confocal imaging and image adjustment of the images after capturing using a fixed integration time of 1 s. The inserts illustrate the fluorescence intensity without image adjustments (note the difference in intensities between the three protocols). The immunostainings were performed with the same secondary antibody dilutions. *Selected nucleus for presentation in insert. Scale bar in **a** is 20 µm (identical for panels **a**–**f**)

The effect of the three pretreatment methods on ethanol-fixed CaSki cells is illustrated in Fig. 5.

The highest fluorescence intensity for 5-mC and 5-hmC was again obtained with the Pepsin/HCl protocol (see inserts in Fig. 5a–f). The impact of the retrieval protocol was most evident for 5-mC, with increasing fluorescence intensity from Citrate via TE to Pepsin/HCl. For 5-hmC, the highest signal intensity increase was found with the TE protocol compared with the Citrate protocol. Supplementary Fig. S1 illustrates the same analyses for the cervical cancer cell lines HeLa and SiHa, which led to essentially the same conclusions as for the analyses of the CaSki cell line.

**Fig. 6:**
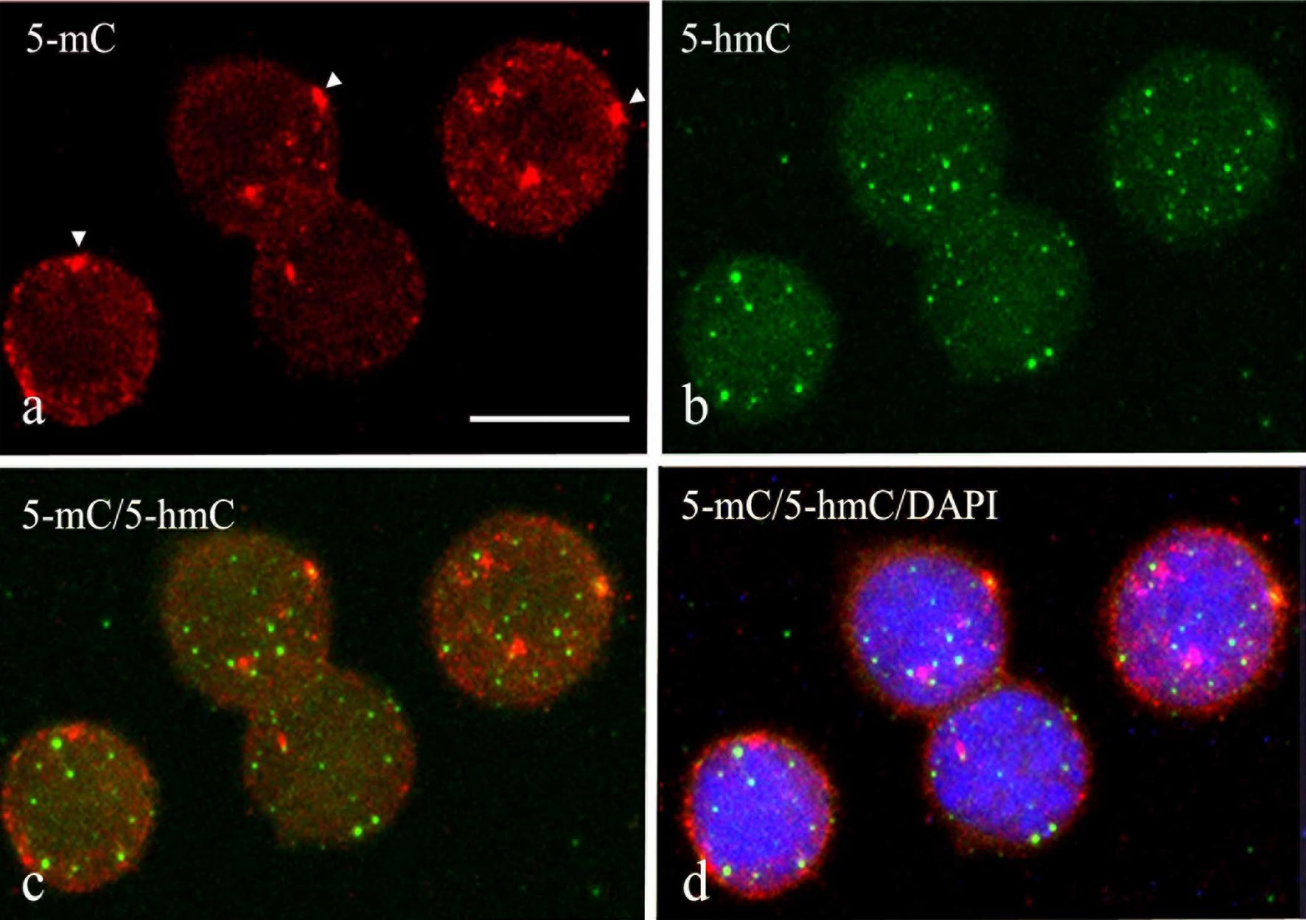
Simultaneous detection of 5-mC and 5-hmC in CaSki cells after the Citrate retrieval method and confocal microscopy imaging. **a** 5-mC in red (Texas Red), **b** 5-hmC in green (FITC), **c** merged image of 5-mC and 5-hmC, and **d** merged image with DAPI included. Note the differences in spot size between 5-mC and 5-hmC and the different frequencies of fluorescent spots for both modifications. Arrowheads point to 5-mC spots/areas at the nuclear periphery. Scale bar in **a** is 20 µm (identical for panels **a**–**d**)

Not only the fluorescence intensity but also the staining patterns changed when comparing the three different retrieval protocols. The fluorescence staining pattern for 5-mC and 5-hmC showed a clear speckled pattern with the TE and Pepsin/HCl protocol (see, for example, Fig. 5c and f and Supplementary Fig. S1c, f, i, and l). At the same time, a concomitant diffuse staining throughout the nuclei was also obtained with all three procedures. This diffuse staining pattern was reduced for representation purposes in the optimized images (after image adjustment) shown in Fig. 5 and Supplementary Fig. S1. To study the localization of 5-mC and 5-hmC relative to each other, in particular the speckled pattern, the modified nucleotides were detected simultaneously in a double-label immunofluorescence approach. Confocal microscopy was used to observe the speckled patterns in a single projection by merging the different confocal levels. Figure 6 illustrates the simultaneous detection of 5-mC and 5-hmC in CaSki using Citrate as the retrieval method. The fluorescence detection of 5-mC (Fig. 6a, red) showed small and large speckles in the nucleus, occasionally being localized close to the periphery of the nucleus (arrowheads in Fig. 6a). For 5-hmC (Fig. 6b, green), mainly small, discrete speckles were observed, with a more uniform spot size and being randomly distributed throughout the nucleus. The merged images for 5-mC and 5-hmC (Fig. 6c, d) demonstrate that the speckles are not overlapping, as concluded from visual inspection and the absence of mixed-colored (orange) speckles. Supplementary Fig. S2 shows similar staining patterns for normal human lymphocytes as compared with the CaSki cells.

### Effect of the pretreatment protocols on 5-mC and 5-hmC detection in chromosome preparations.

**Fig. 7:**
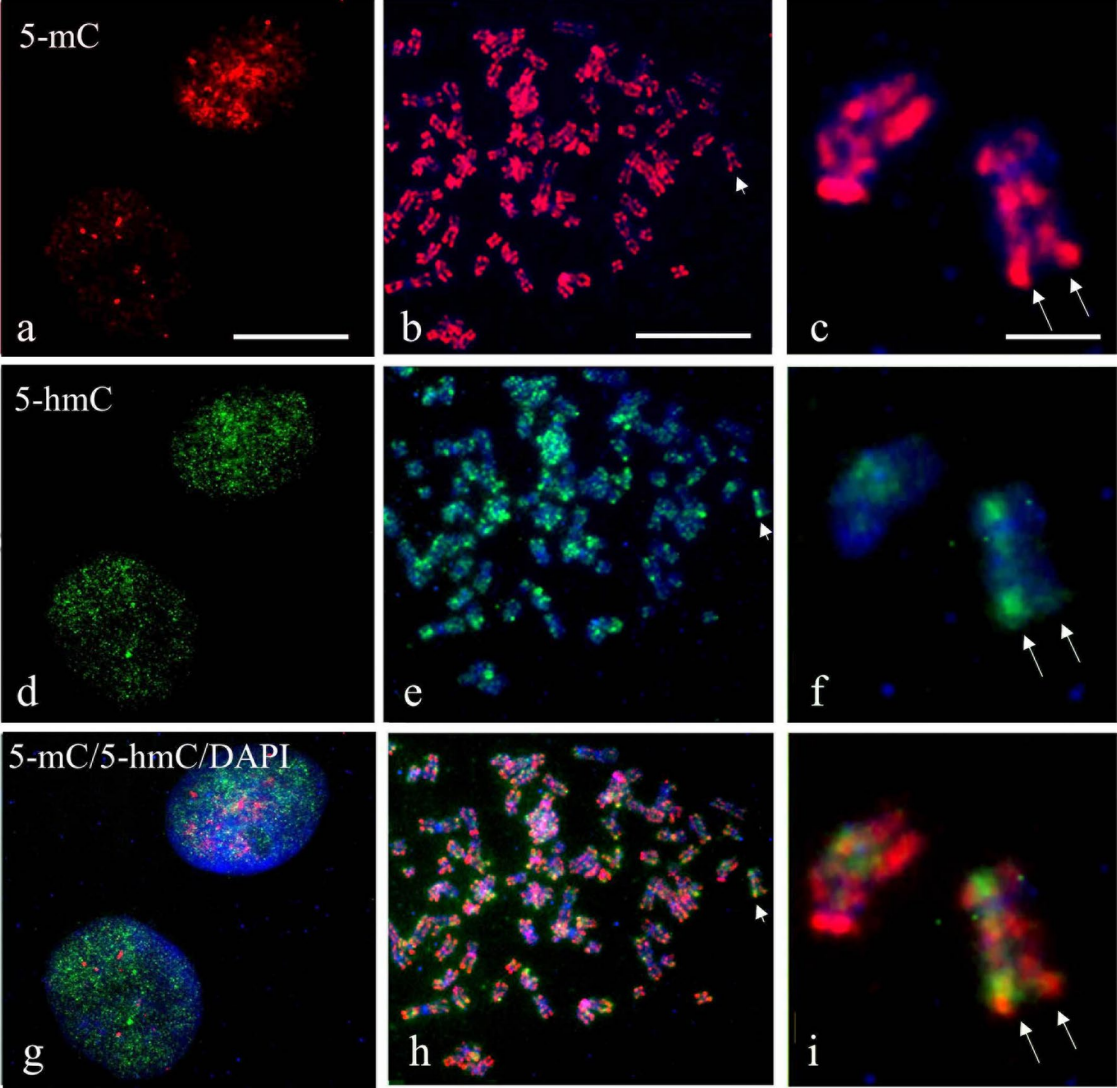
Simultaneous immunofluorescent detection of 5-mC and 5-hmC in CaSki metaphase preparations pretreated with the Citrate retrieval method. **a**, **d**, and **g** CaSki interphase nuclei, **b**, **e**, and **h** a metaphase chromosome plate, and **c**, **f**, and **i** selected metaphase chromosomes. In red (Texas Red) 5-mC, in green (FITC) 5-hmC, and in blue DNA staining using DAPI. The two parallel arrows point to the individual chromatids of a metaphase chromosome. Note for 5-mC the staining of telomeric and centromeric areas, as well as both chromatids and 5-hmC staining of only one of the two chromatids of the chromosome. Scale bar in **a** is 20 µm (identical for panels **a**, **d**, and **g**), and in **b** (identical for panels **b**, **e**, and **h**). Scale bar is 5 µm in **c** (identical for panels **c**, **f**, and **i**)

The three pretreatment protocols were applied to metaphase chromosomes after a hypotonic shock and acetic acid treatment of G2M arrested cultured CaSki cells. During this preparation step of the metaphase chromosomes, interphase cells are also collected on the slides. The subsequent retrieval methods significantly impacted the morphology of the nuclei and metaphase chromosomes. An acceptable morphology for interphase nuclei and metaphase chromosomes could only be obtained with the Citrate protocol. The TE and Pepsin/HCl pretreatment protocols partly disrupted the morphology of the interphase cells and the chromosomes. With the TE protocol, chromosomes sometimes puffed out, while the Pepsin/HCl protocol resulted in the detachment of part of the metaphase chromosomes from the microscope slides. Furthermore, the Pepsin/HCl treatment diminished DNA staining with DAPI. Figure 7 illustrates staining patterns observed for 5-mC and 5-hmC in interphase cells and metaphase chromosomes using the Citrate retrieval protocol.

A speckled pattern is again seen in interphase cells (Fig. 7a, d, and g) and metaphase chromosomes showed an inhomogeneous distribution pattern for both 5-mC and 5-hmC, with alternating positive and negative areas (Fig. 7b, c, e, and f). Centromeric and telomeric regions often exhibited a stronger immunofluorescence staining than the rest of the chromosomes. The 5-mC staining was symmetrically distributed over both sister chromatids within a chromosome (see Fig. 7c; arrows). For 5-hmC, however, we frequently noted that only one of two sister chromatids was labeled, indicating that this epigenetic modification is asymmetrically distributed over the chromatids during or immediately after metaphase (Fig. 7f, and i). In the merged images (Fig. 7g, h, i), the speckled patterns are seen for both 5-mC and 5-hmC in the interphase nuclei, while 5-hmC staining is found only on one of the sister chromatids. To confirm that this phenomenon is not only seen in cancer-derived cells, we also analyzed metaphase chromosomes obtained from normal human lymphocytes. Supplementary Fig. S3 shows that 5-mC staining is again strong in the centromeric and telomeric regions of some of the chromosomes received from a healthy individual (Supplementary Fig. S3**c**; arrowhead and arrow, respectively). The number of speckles and the staining intensity seemed to be reduced compared with what was seen in the cancer cell line. However, 5-hmC was again seen in only one of the two chromatids also in the lymphocyte chromosomes (Supplementary Fig. S3f and i).

## Discussion

Epigenetic modifications, resulting in the activation or silencing of specific genes or gene clusters, often involve the methylation and/or demethylation of deoxycytidine moieties in promotor regions in the DNA. Such modifications have been found in a vast number of genes and have been correlated with a large variety of normal physiological processes, such as cell cycle regulation and cell differentiation. Changes in such methylation and/or demethylation processes have, for example, also been found during aging and (pre)neoplastic progression (Pappalardo and Barra 2021). Since an increasing number of such gene-specific methylation and demethylation events have been published in recent years, we wondered whether or not the immunohisto/cytochemical study of overall methylation and hydroxymethylation in tissues and cells can provide information about, for example, the differentiation status of a specific tissue or cell type. An advantage of such an immunostaining approach is that a heterogeneous distribution of these epigenetic modifications in a given tissue or cell population can be easily recognized, allowing a correlation between the epigenetic switch and its histo-morphological distribution. This is generally not possible with a more integrative test, such as a PCR, enzyme-linked immunosorbent assay (ELISA), or a sequencing approach.

When detecting these epigenetic modifications by means of an immunostaining approach, optimal availability of the antigenic sites for antibody binding is a prerequisite. DNA compactness, proteins binding to the CpG islands, and the fixation procedure may be factors that negatively influence epitope availability. On the contrary, parameters such as low salt, high temperature, acid/alkaline pH, and enzymatic digestion of nuclear proteins may favor the denaturation of DNA and opening of the nuclear structure for epitope recognition.

A comparison of the three retrieval protocols used in the underlying study revealed that the Pepsin/HCl protocol was the most efficient in making 5-mC and 5-hmC available for detection with antibodies and the highest signal intensities were obtained with this method. However, this harsh protocol did affect the nuclear morphology and detectability of DNA using DAPI and diminished inter- and intracellular differences observed with the other two protocols. On the other hand, the Citrate (pH 6.0) and, to a lesser extent the TE (pH 9.0) protocol, left the nuclear organization intact and was superior in visualizing inter- and intranuclear differences in methylation and hydroxymethylation levels. The immunofluorescence intensity obtained with TE (pH 9.0) protocol was higher than when using the Citrate (pH 6.0) method. This difference could result from a more efficient nuclear structure opening under mild alkaline conditions during microwave treatment.

The Pepsin/HCl protocol was initially developed to detect BrdU incorporated into the DNA during the S-phase. To make the labeled DNA optimally accessible for the antibody that recognizes this substituted thymidine in single-stranded DNA, the tissues or cells were treated with DNAse or acid denaturation with 2 M HCl, combined with an enzymatic digestion. These acidic conditions result in the condensation of DNA based on protonation of nuclear phosphate groups, thus limiting enzymatic digestion using Pepsin (Dover and Patel 1994; Schutte et al. 1987). As mentioned before, in our study, the Pepsin/HCl method resulted in DNA loss as detected by reduced DNA staining. Loss of morphology was particularly evident in ethanol-fixed cells and metaphase preparations. In addition, the reduced DNA staining by the intercalating DNA dye DAPI was observed in all specimens, probably due to hydrolysis of the double-stranded DNA, leading to denaturation and blocking the intercalation of DAPI.

### Inter- and intranuclear differences for 5-mC and 5-hmC

A fluorescence intensity range of several magnitudes was typical for both 5-mC and 5-hmC in FFPE normal squamous epithelium. This large range resulted mainly from the intensity differences detected in cells belonging to the different compartments of the epithelium, e.g., the basal/parabasal, intermediate, and superficial layers. Also, the methylation and hydroxymethylation levels can alternate in neighboring cells within a single compartment or between different cellular compartments. It can be debated whether or not these differences are a direct consequence of differences in methylation and hydroxymethylation levels or result from differences in DNA compactness, in which case the pretreatment protocol dictates the availability of the modified nucleotides for immunohistochemical detection. Since heterogeneity is detected with all protocols, it is most likely that intrinsic differences in methylation and hydroxymethylation levels cause these observed heterogeneous staining patterns.

The observed heterogeneity makes a comparison with biochemical assays measuring methylation and hydroxymethylation levels difficult. Mixing hyper(hydroxy)methylated DNA with hypo(hydroxy)methylated DNA will be averaged out in a biochemical assay. Only an immunocytological or immunohistological analysis enables the correlation of such a heterogeneous distribution with histo-morphological features of a tissue.

### Distribution of 5-mC and 5-hmC fluorescence on the chromosomes

The nuclei of FFPE squamous epithelium and of the cell lines, and the intact nuclei in the metaphase preparations showed a punctate 5-mC fluorescent nuclear distribution pattern, occasionally localized at the periphery of the nuclei. The metaphase chromosomes often showed accumulations of 5-mC fluorescent staining in the centromeric and telomeric regions of specific chromosomes. On top of these intensely stained areas, a diffuse pattern throughout the nucleus and chromosome was observed. These observations support the high level of methylation found in heterochromatin (containing silenced genes) and lower levels of methylation observed in euchromatic areas in the nucleus (Pappalardo and Barra 2021). The localization of 5-hmC, which has not yet been described in detail in the literature, showed that the fluorescent patches for 5-hmC are smaller than those for 5-mC, they are more evenly distributed over the nuclei, and their frequency showed to be higher. Our study showed that the fluorescent patterns for 5-mC and 5-hmC are not overlapping, which does not exclude that at the molecular level, within small chromosomal domains, the 5-mC and 5-hmC can colocalize.

In the individual metaphase chromosomes, differences in staining intensity for 5-mC and 5-hmC are recognized over the length of the chromosome, which cannot be directly correlated to DNA compactness. The methylation (5-mC) pattern is copied from the parent strand to the daughter strand during somatic cell division and is maintained during DNA replication by conversion of hemi-methylated sites back to symmetrically methylated molecules by DNA methyltransferase DNMT1 to prevent loss of the mark (Petryk et al. 2021). The 5-mC distribution, therefore, is stable with chromosomes being methylated in R-bands and the heterochromatic regions 1q12, 9q12, and 16q11.2. In cancer cells, this pattern is altered and methylation in repetitive elements can even influence cell division (Nishiyama and Nakanishi 2021). Over-methylation during the cell cycle has been reported and the cell actively demethylates the genome during G0/G1 to restore the methylation patterns to the correct state (He et al. 2021). The TET enzymes that are responsible for the formation of 5-hmC play a role in this demethylation process. The 5-hmC pattern is more than just copied during cell division (Shen and Zhang 2013). Earlier studies (Kubiura et al. 2012) have revealed a sharp difference in the levels of 5-hmC in the two opposite DNA strands of a given chromosome, and a chromosome-wide cell-to-cell variability in mammalian cells. Bachman et al. (2014) demonstrated that 5-hmC forms slowly during the first 30 h following DNA synthesis, while DNA methylation occurs immediately during replication. This asymmetric distribution and difference in chromosome hydroxymethylation was also recognized in our study of metaphase chromosomes from the HPV-positive human cancer cell line and from normal human lymphocytes.

We conclude that immunohistochemical detection of 5-mC and 5-hmC enables the direct correlation of these DNA modifications with histo-morphological features in heterogeneous tissues and cell populations, but is influenced by different pretreatment protocols that must be carefully chosen to allow an appropriate interpretation of these epigenetic switches. While TE and Pepsin/HCl were most efficient for the immunochemical detection of 5-mC and 5-hmC with respect to signal intensity, the Citrate protocol did preserve nuclear morphology and enabled the visualization of intra- and internuclear epigenetic differences in FFPE tissue and cell culture samples. This counts for single-color bright-field microscopy and single- and double-label fluorescence detection using confocal and non-confocal microscopy. The simultaneous visualization of 5-mC and 5-hmC showed intra- and internuclear differences in staining intensity and localization for these epigenetic modifications that point to intrinsic differences in methylation and hydroxymethylation levels.

## Supplementary Information

Below is the link to the electronic supplementary material.

Supplementary file1 (PDF 590 KB)

## Data Availability

The study includes original data, A.H.N. Hopman confirms that he had full access to all the data in the study and takes responsibility for the integrity of the data and the accuracy of the data analyses.
