# Supplementary material for: Impact of antigen retrieval protocols on the immunohistochemical detection of epigenetic DNA modifications
Source: Histochem Cell Biol. 2023 Apr 3;159(6):513–26. doi: 10.1007/s00418-023-02187-4 (PMC10247850; doi:10.1007/s00418-023-02187-4)
Supplement: Supplementary file 1 — Supplementary file1 (PDF 590 KB) [file 418_2023_2187_MOESM1_ESM.pdf]

# Impact of antigen retrieval protocols on the immunohistochemical detection of epigenetic DNA modifications

Running title: Immunohistochemistry of 5-(hydroxy)methylcytosine

Jobran M. Moshi[1, 2], Monique Ummelen[1], Jos L. V. Broers[1], Frans C.S. Ramaekers[1], Anton H N Hopman[1 *]

[1]Department of Molecular Cell Biology, GROW-School for Oncology & Reproduction, Maastricht University Medical Center, Maastricht, the Netherlands.

[2]Department of Medical Laboratory Technology, Faculty of Applied Medical Sciences, Jazan University, Jazan, Kingdom of Saudi Arabia.

*Corresponding author:

Anton Hopman, Ph.D.
Department of Molecular Cell Biology,
Maastricht University Medical Center,
P.O.Box 616, 6200 MD Maastricht, the Netherlands.
Mobile: +31 646139090.

E-mail: hopman@maastrichtuniversity.nl

Keywords: uterine cervix, squamous epithelium, cultured cells, chromosomes, 5-methylcytosine, 5-hydroxymethylcytosine, immunohistochemistry, antigen retrieval.

Supplemental Figure S1

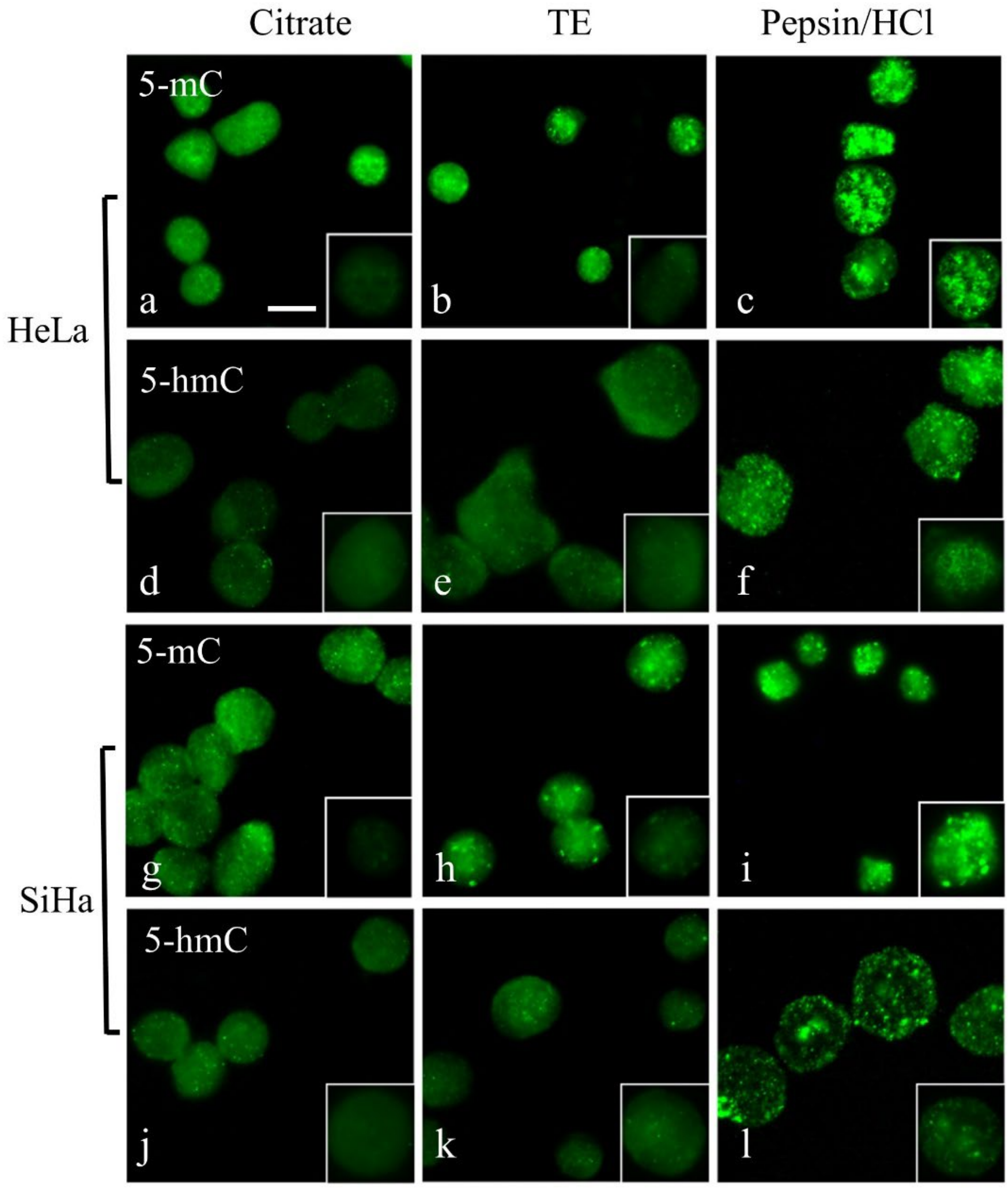


Comparison of immunofluorescent staining of 5-mC and 5-hmC in HeLa and SiHa cells using the Citrate, TE and Pepsin/HCl retrieval methods.

**a-l**) Visualization of 5-mC and 5-hmC (both in green) with non-confocal imaging and adjustment of the images collected using a fixed integration time of 1 sec. The difference in fluorescence intensity is illustrated in the inserts that show the recorded fluorescence images without adjustments. The immunostainings were performed with the same secondary antibody dilution. Scale bar in **a** indicates 20 µm (identical for panels **a-l**).

Supplemental Figure S2

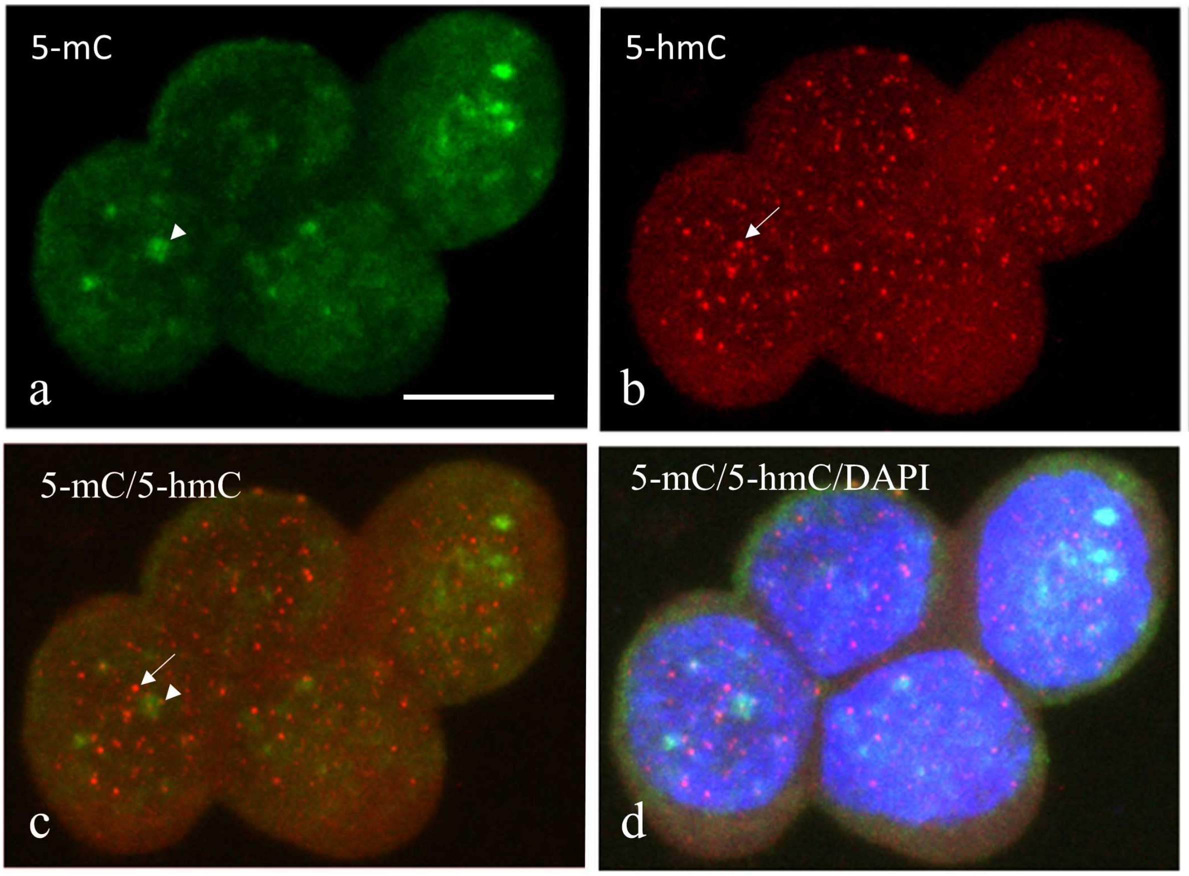


Simultaneous detection of 5-mC and 5-hmC in ethanol-fixed normal human lymphocytes pretreated with the Citrate protocol and imaged with confocal microscopy.

**a**) 5-mC in green (FITC), **b**) 5-hmC in red (Texas Red), **c**) merged image of 5-mC and 5-hmC and **d**) merged images with DAPI as counterstaining. Note the difference in spot size between 5-mC and 5-hmC and the difference in the frequency of fluorescent spots for both modifications. The arrowhead points to a large fluorescent spot for 5-mC (**a**) and the arrow to a small spot for 5-hmC (**b**); the spots are not overlapping but are close to each other (**c**). Scale bar in **a** indicates 20 µm (identical for panels **a**-**d**).

Supplemental Figure S3

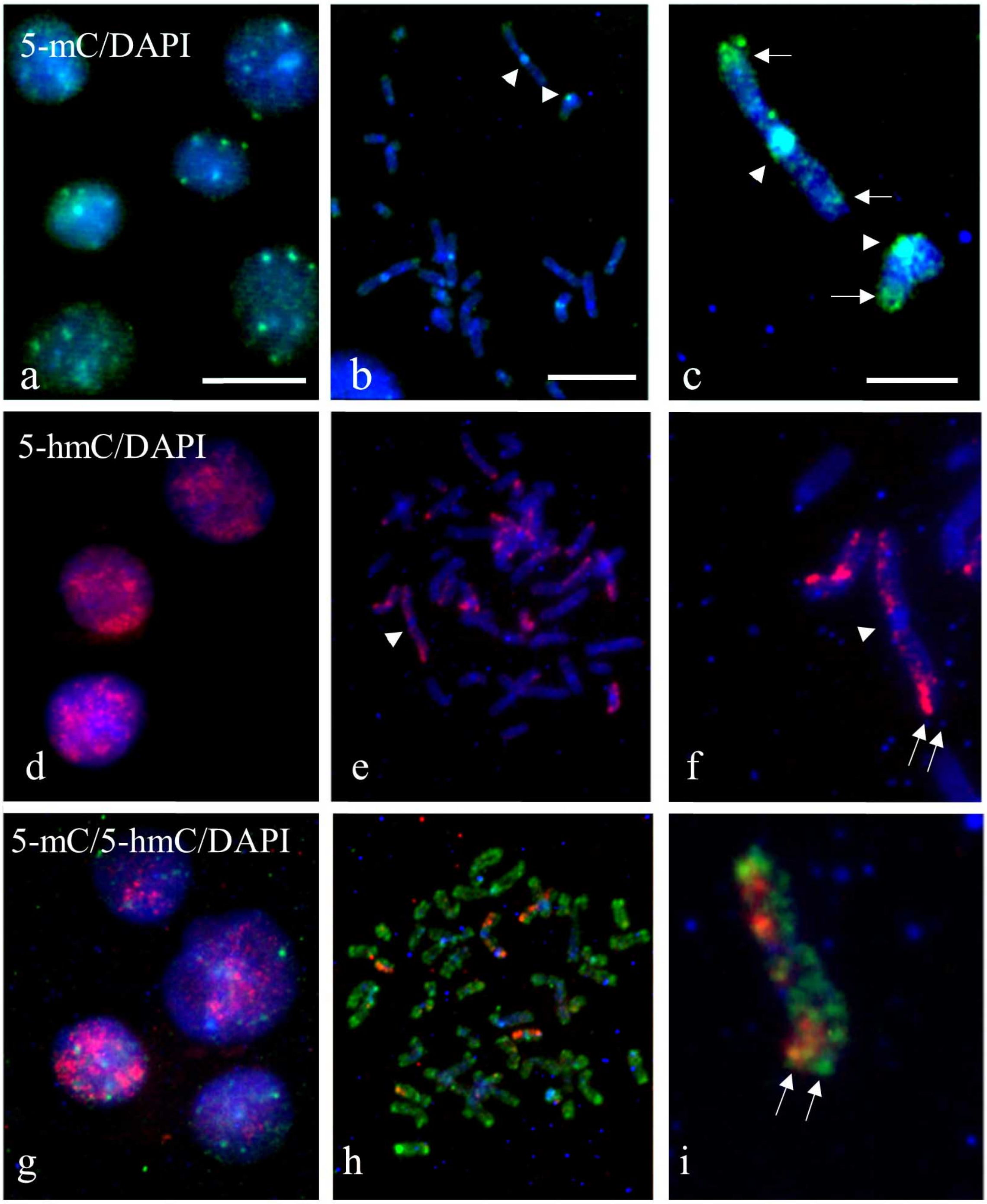


Simultaneous immunofluorescent detection of 5-mC and 5-hmC in TE-treated lymphocyte metaphase preparations.

**a**, **d** and **g**) lymphocyte interphase nuclei, **b**, **e** and **h**) metaphase chromosome plate and **c**, **f**, and **i**) selected metaphase chromosomes. In green (FITC; **a-c**, **g-i**) 5-mC, in red (Texas Red, **d-i**) 5-hmC and in blue DNA staining using DAPI (in all images). In **c**, **f** and **i** selected metaphase chromosomes (see arrowheads in **b** and **e**) show the telomere (arrows) and

centromere (arrowheads) staining for 5-mC in both chromatids. In **c** the intense 5-mC staining is recognized in the 1q12 area, an AT-rich band that is strongly stained with DAPI. The positive staining of 5-hmC in only one chromatid can be clearly seen in **f** and **i** (see parallel arrows). Scale bar in **a** indicates 20 μm (identical in panels **a**, **d** and **g**) and **b** (identical in panels **b**, **e** and **h**) and 5 μm in **c** (identical in panels **c**, **f** and **i**).
